# Continuous joint velocity estimation using CNN-based deep learning for multi-DoF prosthetic wrist for activities of daily living

**DOI:** 10.3389/fnbot.2023.1185052

**Published:** 2023-09-07

**Authors:** Zixia Meng, Jiyeon Kang

**Affiliations:** ^1^Mechanical and Aerospace Engineering, School of Engineering and Applied Sciences, University at Buffalo, Buffalo, NY, United States; ^2^Electrical Engineering, School of Engineering and Applied Sciences, University at Buffalo, Buffalo, NY, United States; ^3^School of Integrated Technology, Gwangju Institute of Science and Technology, Gwangju, Republic of Korea; ^4^AI Graduate School, Gwangju Institute of Science and Technology, Gwangju, Republic of Korea

**Keywords:** prosthetic control, deep learning, training strategy, surface electromyography, activities of daily living

## Abstract

**Introduction:**

Myoelectric control of prostheses is a long-established technique, using surface electromyography (sEMG) to detect user intention and perform subsequent mechanical actions. Most machine learning models utilized in control systems are trained using isolated movements that do not reflect the natural movements occurring during daily activities. Moreover, movements are often affected by arm postures, the duration of activities, and personal habits. It is crucial to have a control system for multi-degree-of-freedom (DoF) prosthetic arms that is trained using sEMG data collected from activities of daily living (ADL) tasks.

**Method:**

This work focuses on two major functional wrist movements: pronation-supination and dart-throwing movement (DTM), and introduces a new wrist control system that directly maps sEMG signals to the joint velocities of the multi-DoF wrist. Additionally, a specific training strategy (Quick training) is proposed that enables the controller to be applied to new subjects and handle situations where sensors may displace during daily living, muscles can become fatigued, or sensors can become contaminated (e.g., due to sweat). The prosthetic wrist controller is designed based on data from 24 participants and its performance is evaluated using the Root Mean Square Error (RMSE) and Pearson Correlation.

**Result:**

The results are found to depend on the characteristics of the tasks. For example, tasks with dart-throwing motion show smaller RSME values (Hammer: 6.68 deg/s and Cup: 7.92 deg/s) compared to tasks with pronation-supination (Bulb: 43.98 deg/s and Screw: 53.64 deg/s). The proposed control technique utilizing Quick training demonstrates a decrease in the average root mean square error (RMSE) value by 35% and an increase in the average Pearson correlation value by 40% across all four ADL tasks.

## 1. Introduction

The human upper limb function is crucial to perform daily living activities. The loss of one or both arms causes severe disability that greatly affects a person's ability to perform essential daily activities (Kuiken et al., 2009). To date, there are nearly two million people living with limb loss in the United States, with ~41,000 individuals suffering from major upper limb amputations (Atzori and Müller, 2015). The number of individuals with amputation is increasing, resulting in a significant rise in health care costs. In 2009, hospital costs associated with amputation totaled more than 8.3 billion dollars (Semasinghe et al., 2019). As a result, the development of upper-limb prosthetic devices is considered to be crucial in helping amputees adapt to daily activities and reintegrate into society.

In order to restore the upper limb function of amputees, the development of myoelectric prosthesis started in the early 1940s (Kobrinskiy, 1960; Popov, 1965). A myoelectric prosthesis is electrically-powered, utilizing the electrical signals generated from some flexor and extensor muscles of the residual limb, which are surface electromyography (sEMG) signals that reflect the user's intention. To date, almost all commercial electric prostheses use a “direct myoelectric control” approach, where each direction of a motor in a prosthetic joint or the opening/closure of a specific grasp type in a robotic hand is controlled by a specific muscle. The myoelectric controller often uses the on-off method using a pre-defined threshold, but all commercial manufacturers also provide proportional control that can provide essentially continuous output to the active DoF of the prosthetic system (Fougner et al., 2012). To actuate multiple active degrees of freedom prosthetic devices, state machine technique has been suggested, which employs two sEMG signals to operate a single joint but also permits switching between other joints by co-activation of both muscles (Vujaklija et al., 2016). For example, SSSA-MyHAND (Controzzi et al., 2017) used state-machine, which switched to various grasps such as lateral, bi-directional, power, hook, pointing up and down by co-activation of both muscles. The state-machine complexity increases significantly when the number of prosthetic joints increases (Resnik et al., 2018) and it lacks the capability of simultaneous control of multiple DoFs which hinders the dexterity of the hand movement during daily living tasks.

Pattern recognition has been suggested and widely explored for the past few decades (Hargrove et al., 2007). Based on sEMG activation patterns, the amplitude of sEMG was used to decode the information and transfer the instructions to the motor, that could identify the user's intended hand and wrist motions (Scheme and Englehart, 2011; Parajuli et al., 2019). Statistical methods such as LDA (Linear Discriminant Analysis) and SVM (Support vector machine) were used to classify user intention with feature extraction, which were clinically tested on several amputee trials (Al-Timemy et al., 2013; Stango et al., 2014). For neural-based models, ANN (Artificial neural network) and MLP (Multilayer perceptron) were one of the initial deep learning algorithms researchers explored (Kawasaki et al., 2014). In comparison to traditional methods, these models were easily trainable and have the capability of modeling with non-linear data (Ahmad et al., 2011). Recently, Tam et al. (2021) designed a gesture recognition system using a CNN for myoelectric hand prosthesis control, in which the user could be able to monitor the gesture recognition output in real time. This pattern recognition-based classification method could only support discrete movement classification, which was rather non-intuitive compared to the natural way of controlling hands' pose (Yang et al., 2022).

To overcome the limits of classification approaches, several researchers have used deep learning techniques to control hand movements with regressions. Bao et al. (2021b) proposed the regression supervised domain adaptation (SDA) for estimating wrist angles using sEMG data. This study investigated the domain-shifting problem of the model when handling new subjects by categorizing the dataset of each subject as either the source or target domain and generating pairwise samples instead of single ones. A specific loss function, discrepancy loss, was also introduced for better description of the data. Stival et al. (2018) combined and IMU (Inertial Measurement Unit) features for the control of prosthetic devices. However, the study by Bao et al. was limited to simple wrist flexion/extension movements, while Stival et al.'s study was based on an online database and only presented two movements (flexion of three fingers or flexion of the wrist), which had the best performance.

**Figure 1:**
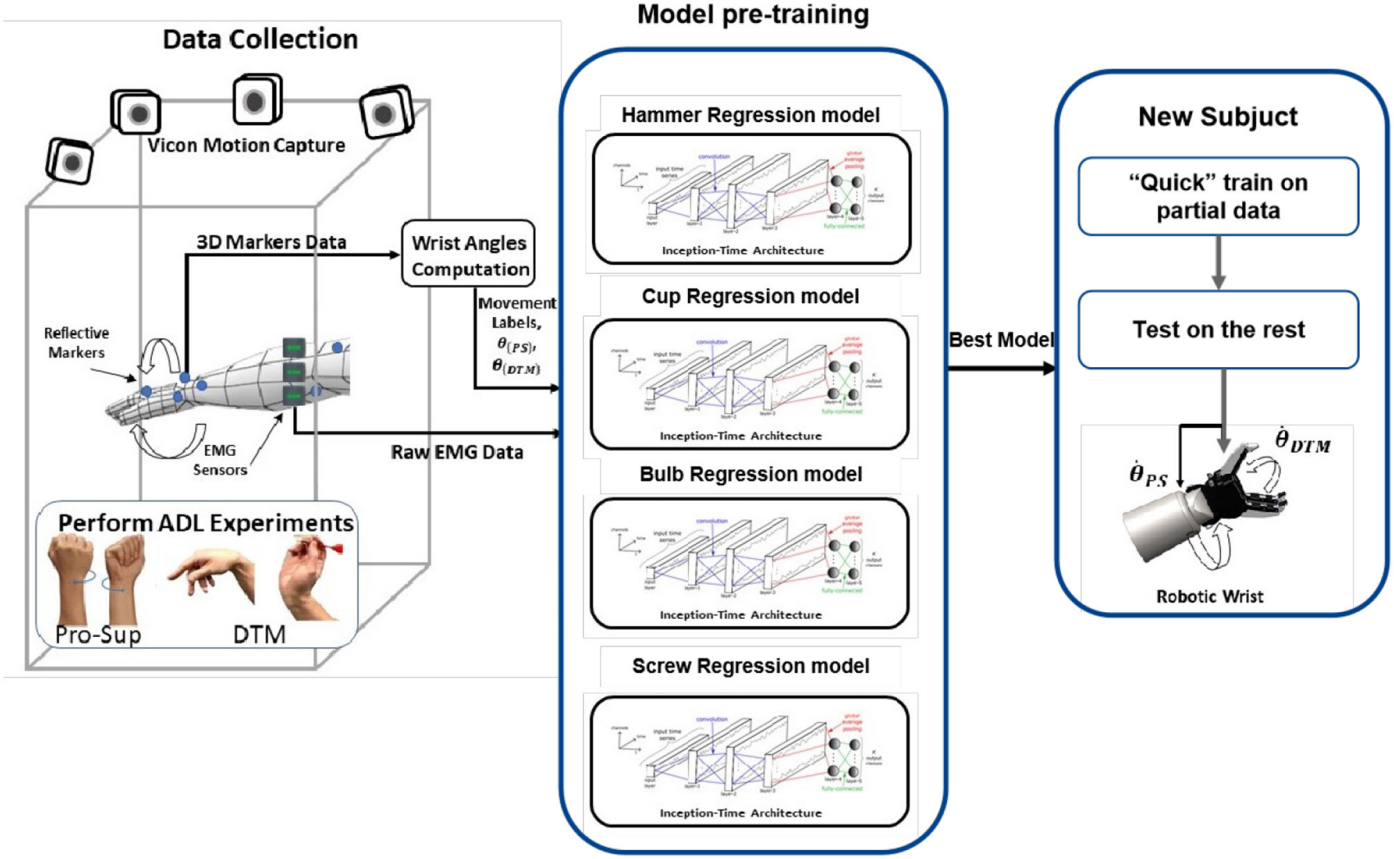
Overview of the proposed method. This multi-DoF controller will estimate the angular speed for pronation/supination (PS) and dart-throwing movements (DTM) with a training strategy.

In this study, to overcome the limitations of existing methods, a CNN-based wrist controller using a regression model is proposed and evaluated based on real-life ADL data. The proposed controller continuously estimates the wrist angle velocity from sEMG sensors placed on the participant's forearm, enabling continuous control of a multi-DoF prosthetic wrist in a more natural way. The model was trained using data collected while participants performing ADL tasks that focused on pronation-supination and dart-throwing-motion of the wrist. To increase the robustness of the model, ADL tasks were conducted to collect movement data at different heights. To use this model by a new participant within a short time, a method utilizing Pre-training and Quick training data is also suggested. This method can be used by participants within the existing data set to reduce the retraining time, as fast training is frequently required for amputee participants due to donning-doffing, muscle fatigue, or contamination (e.g., sweat; Ameri et al., 2020). An overview of the proposed method is shown in Figure 1. The results varied depending on the characteristics of the tasks. For example, tasks with dart-throwing motion showed smaller RSME values (Hammer: 6.68 deg/s and Cup: 7.92 deg/s) compared to tasks with pronation-supination (Bulb: 43.98 deg/s and Screw: 53.64 deg/s). The proposed control technique utilizing Quick training demonstrated a decrease in the average root mean square error (RMSE) value by 35% and an increase in the average Pearson correlation value by 40% across all four ADL tasks.

## 2. Data collection

The study was approved by the Institutional Review Board of University at Buffalo. Participants provided written consent prior to the experiment. Only individuals with fully functioning biological arms and unrestricted arm movement were included in the study. And, for the current feasibility test, we recruited only right-handed participants to ensure homogeneous data. Participants included 24 healthy individuals. Their average age, height, and weight were 25.38 ± 3.00 years, 171.74 ± 8.40 cm, and 69.90 ± 14.67 kg, respectively. All participates were right-handed.

### 2.1. Sensor system

**Figure 2:**
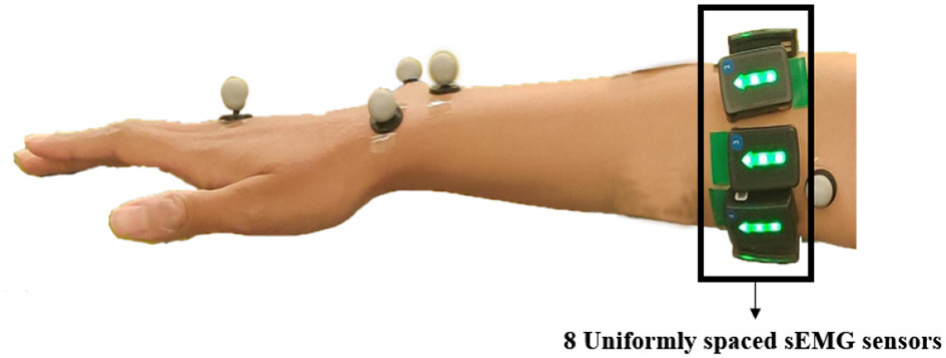
sEMG sensors were placed beneath the elbow, uniformly spaced from each other.

The Trigno^Ⓡ^ Wireless Biofeedback System (Delsys, MA) is a device designed to make and biofeedback signal detection reliable and easy. The system transmits signals from Trigno Avanti^TM sensors to a receiving base station using a time-synchronized wireless protocol that minimizes latency in data transmission across sensors. In this study, eight sensors were placed around the forearm near the elbow to capture muscle signals during experiments, as depicted in Figure 2. The sEMG sensor data was sampled at 2,000 Hz. Ten Vero motion capture cameras (Vicon, UK) were used to capture the movements of the participants. A total of nine markers were placed on the upper body and were divided into four different body segments (Fazil et al., 2022).

### 2.2. Experimental task

**Figure 3:**
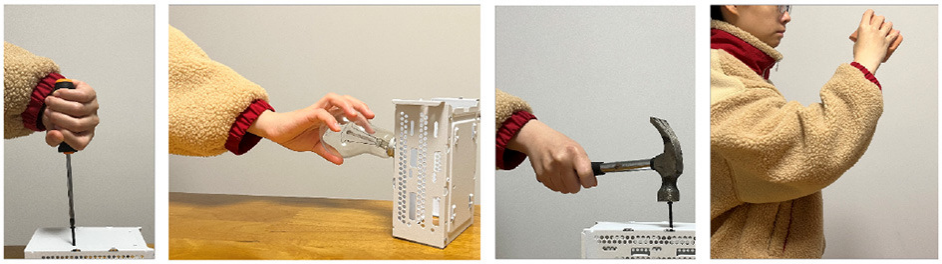
The activities of daily living (ADL) tasks are trained/tested through the Screw rotation, Bulb twisting, Hammering, and Cup drinking (from left to right).

Four representative activities of daily life were specifically chosen for the experiment focusing on pronation-supination (PS) movement or dart-throwing movement (DTM). Specifically, PS and DTM were chosen for our prosthetic emulator in Poddar et al. (2021) and Poddar and Kang (2022). The Bulb twisting task and the Screwdriver task were designed for PS movements, and the Hammering task and the Cup drinking task were designed for DTM, as depicted in Figure 3. In each experiment, the participant started the tasks once all the sensors and markers had been placed. For each experiment, the participant was provided with different tools set up on a table in front of them. For the Bulb Twisting task, a custom-made board with a bulb socket fitted in parallel to the participant was placed at the edge of the table, and a bulb was placed within reach to its right. For the Screwdriver task/Hammering task, a steel panel with a nail in the center was fixed by a clamp and placed at the edge of the table, while the screwdriver/hammer was placed within reach to its right. The nail was placed ~2 cm above the table. For the Cup drinking task, a paper cup was placed in front of the center of the participant's body on the table within reach.

For each trial, the procedure was as follows: First, the participant started from the T-pose position which stretches the arm shoulder height with palms facing down and feet on designated marks on the floor. The participant's toes were ~40 cm away from the edge of the table, with the distance adjusted based on the reach range of each individual. Recording began after a voice cue. After 2 s of recording, the participant was visually/orally prompted to begin. In the Screwdriver/Hammering/Cup Drinking task, the participant reached forward to pick up the screwdriver/hammer/cup and performed the screwing/hammering/drinking action 10 times. The procedure for the Bulb twisting task was slightly different. The twisting was performed 10 times in a clockwise direction and 10 times in a counterclockwise direction. After the participant completed the final movement, the tools were returned to the initial position on the table.

For each activity of daily living task, the trial was repeated three times by incrementally increasing the height of the table. The height of the table for the first trial started at 78.5 cm and increased by 5 cm each time, ending at 88.5 cm. A verbal cue was given before each trial to start. The participant was instructed to perform the movements at a consistent speed to maintain uniformity and integrity of data. A practice trial was conducted prior to the recording sessions to familiarize the participant with the steps involved in each trial. Participants performed four tasks sequentially in random order.

### 2.3. Data set generation

**Figure 4:**
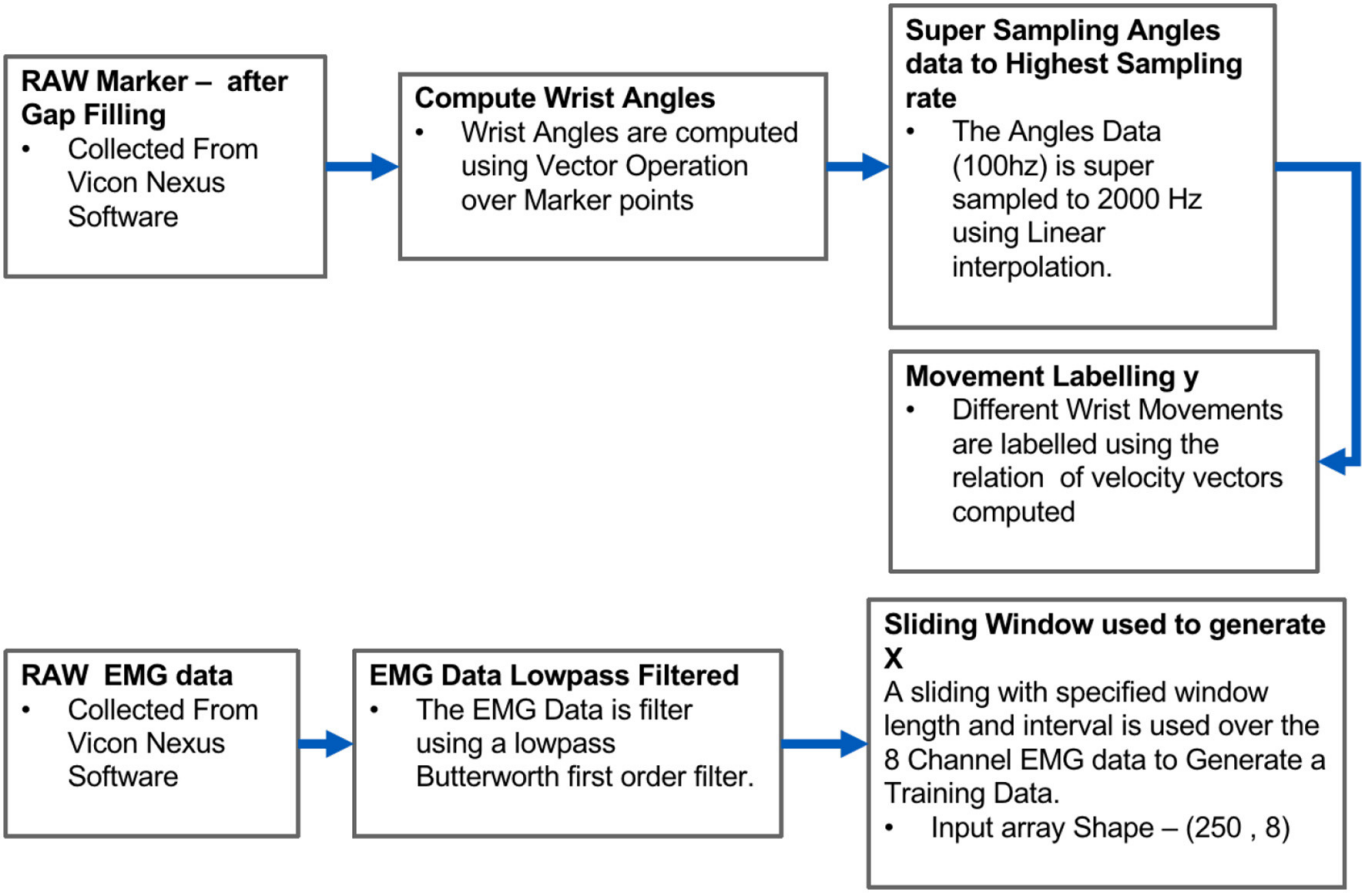
Raw data processing pipeline for features and labels.

The data collection system consisted of a motion capture system, eight Delsys wearable sensors, a height-adjustable table, and four sets of tools for conducting experiments. In the experiment of this study, upper limb motion is measured using ten motion capture cameras and sEMG data were collected from eight wireless Trigno sensors. In the present ADL tasks, two angles were calculated: the pronation-supination (PS) angle and the dart-throwing motion (DTM) angle. These angles were calculated by constructing pairs of vectors within the markers in 3D space and computing the angle between them as in Fazil et al. (2022). As shown in Figure 4, the sEMG data were first filtered using a low-pass Butterworth first-order filter at 1 Hz. To generate feature data, the filtered data from eight sensors were cut into segments using a sliding window. The length of the window was set to 250 frames, which corresponds to 125 ms, with an overlap of 240 frames. The resulting feature data had a shape of (250, 8).

## 3. Deep learning wrist controller

### 3.1. Inception-time model

Hierarchical Vote Collective of Transformation-based Ensembles (HIVE-COTE; Lines et al., 2016) recently emerged as one of the most popular methods for Time Series Classification tasks; Such method is a meta-ensemble built on several classifiers, including Time Series Forest, Shapelet Transform Classifier, and KNN-based classifiers. Although this algorithm has achieved outstanding performance on the benchmark datasets, it suffers from *O*(*n*^2^·*T*^4^) time complexity. Recently, Ismail Fawaz et al. (2020) introduced a deep Convolutional Neural Network (CNN), called Inception-Time, which not only outperforms the accuracy of HIVE-COTE but is also substantially faster while the complexity of Inception-Time increases almost linearly with an increase in the time series' length. The high accuracy and scalability of Inception-Time make it an ideal candidate for system development. In this study, we adapted the Inception-Time model to handle regression tasks.

- The fully-connected layer at the end of the network is substituted by a fully connected dense layer.
- The loss function is changed to a mean-square-error function.
- In each Inception module, kernel sizes and the numbers of filters are selected to fit the study.

### 3.2. Quick training strategy

**Figure 5:**
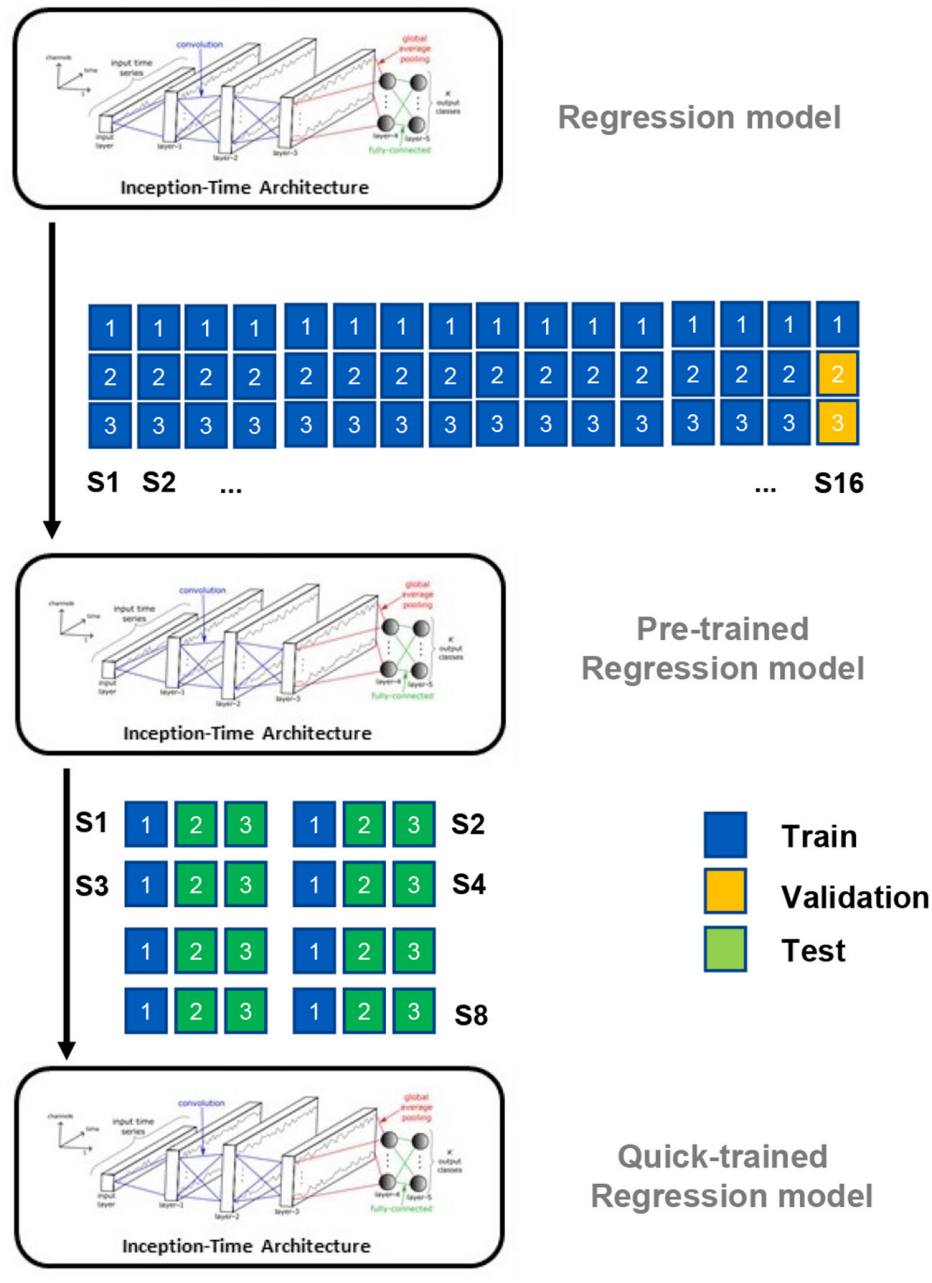
Proposed training strategy including Pre-training process and Quick training process.

As depicted in Figure 5, a unique training strategy is proposed. In this study, 24 participants performed three trials. The data was divided into four parts: pre-training group, model selection group, “Quick training” group, and test group. The pre-training group consisted of all trials of the first 15 individuals and the first trial of the 16th participant's three trials. The data in this group was used to initially train the modified Inception-Time model. The remaining two trials of the 16th participant were used as the validation set, and the model with the best performance, as measured by Pearson Correlation, was selected. The remaining data from the eight participants were considered new subjects, as they were unseen by the selected model. For each participant, the first trial was used for “Quick training,” and the model was evaluated on the rest two trials.

For the implementation of the Inception-Time model and Quick training, Python 3.0 was used to design the wrist controller. The NumPy Python library is frequently used for scientific computing operations. The model was built on TensorFlow 2.5.0. Tools which was used for generating labels, normalization, and performance evaluations in Python. Most parts of our programs were computed on an NVIDIA GeForce RTX 3080 10G GPU.

In the present study, four different models were created for each task. The tasks could be divided into pronation-supination based Bulb and Screw tasks and dart-throwing-motion based Hammer and Cup tasks. The input of models was set in the form of (250, 8), which means the length of the sliding window is 250 frames (125 ms), and eight-channel signals were collected from eight sEMG sensors. Besides the Butterworth filter mentioned before, a scaler was used to normalize the data when generating features from the data. Same scaler was also applied to the data of the validation group, “Quick training” group, and test group.

### 3.3. Performance metrics

In this study, two common measures are used for numerical evaluations: Root Mean Square Error (RMSE) and Pearson correlation (PC), with following formulas. θ_*i*_ represents the true angle (PS angel or DTM angle) at time frame *i*, while $\overset{•}{\theta_{i}}$ represents the true joint velocity at time frame *i*. $\hat{\overset{•}{\theta }}$ stands for the predicted joint velocity, and $\bar{\left(\cdot \right)}$ as the mean value of (·). The number of total time frames is denoted as *n*.


$$\text{RMSE}=\sqrt{\frac{\sum_{i=1}^{n}\|\hat{\dot{\theta_{i}}}-\dot{\theta_{i}}{\|}^{2}}{n}}$$

$$\text{Pearson Correlation}=\frac{\sum_{i=1}^{n}\left(\hat{\dot{\theta_{i}}}-\bar{\hat{\dot{\theta }}})(\dot{\theta_{i}}-\bar{\dot{\theta }}\right)}{\%\sqrt{\sum_{i=1}^{n}{\left(\hat{\dot{\theta_{i}}}-\bar{\hat{\dot{\theta }}}\right)}^{2}}\sqrt{\sum_{i=1}^{n}{\left(\dot{\theta_{i}}-\bar{\dot{\theta }}\right)}^{2}}}$$

where Pearson Correlation is a measure of linear correlation between two sets of data. It is essentially a normalized measurement of the covariance, such that the result always has a value between −1 and 1.

## 4. Result

**Figure 6:**
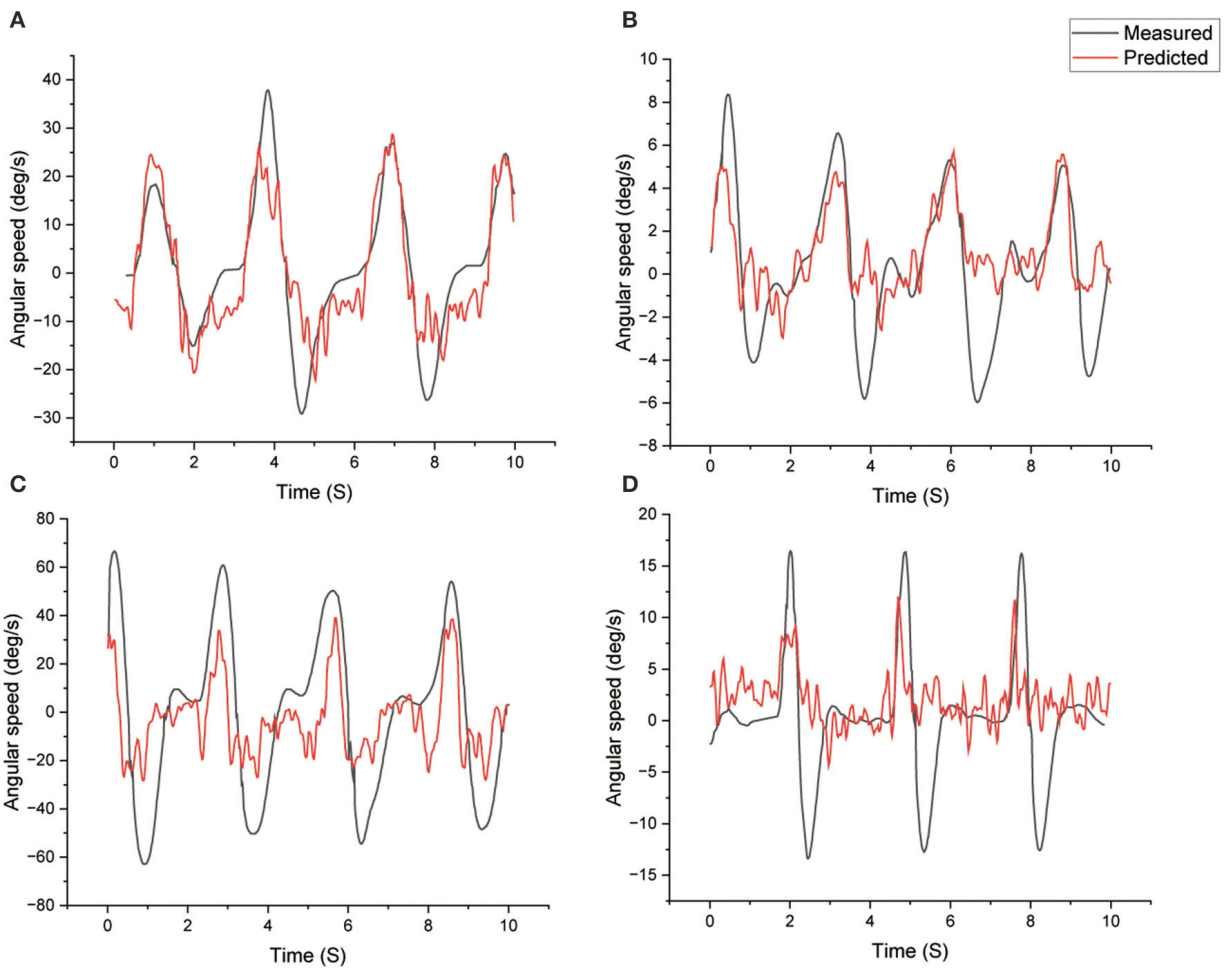
Comparison between measured (true) and predicted angular velocity of four different tasks with Quick training. **(A)** Screw, **(B)** cup, **(C)** bulb, **(D)** hammer.

The comparisons between the measured and predicted data with Quick training of four different tasks are depicted in Figure 6. The data shows the data fit better for the positive values compared to the negative angular speed in general. The Screw and Bulb task follows the true value better. The Cup and Hammer task has smaller range of angular speed compared to Screw and Bulb tasks.

The Bulb task used a model with a depth of 5, which means five Inception blocks are used. In each block, there are three convolutional layers with kernel sizes of 64, 16, and 4, respectively. The number of filters is 128. The numbers of epochs for the pre-training and “Quick training” part are both set as 30. When the model is pre-training, optimizer Adam (adaptive moment estimation) is used with a learning rate starting as 1e-3 and other parameters as default. The learning rate is decayed to half of its original value every 10 epochs. On data from the pre-training group, the selected model has RMSE of 19.723 deg/s, and Pearson Correlation of 0.669. On data from the validation group, the selected model has RMSE of 21.123 deg/s, and Pearson Correlation of 0.628. For the Screw task, an eight-depth model is utilized, which employs eight Inception blocks. Each block is composed of three convolutional layers with kernel sizes of 64, 16, and 4, respectively. The number of filters used is 128. Pre-training is done for 40 epochs, while “Quick training” is done for 30 epochs, using the Adam optimizer as before. The pre-training group achieved RMSE of 9.467 deg/s and Pearson Correlation of 0.849. On the validation group data, the selected model achieved RMSE of 25.265 deg/s and Pearson Correlation of 0.727.

For the Hammer task, a model with a depth of 4 is employed, utilizing four Inception blocks. Each block contains three convolutional layers with kernel sizes of 64, 16, and 4, respectively. The number of epochs for pre-training and “Quick training” is set to 30, and the Adam optimizer is used as before. The selected model achieved RMSE of 5.679 deg/s and Pearson Correlation of 0.817 on the pre-training group data. However, on the validation group data, the selected model achieved RMSE of 5.385 deg/s and Pearson Correlation of 0.166. As for the Cup task, a model with a depth of 3 is used, employing three Inception blocks. Each block consists of three convolutional layers with kernel sizes of 128, 32, and 8, respectively. The number of epochs for pre-training and “Quick training” is set to 30, and the Adam optimizer is used as before. Unfortunately, during the second trial of Subject 2, the sEMG sensors disconnected from the software, so the entire set of Subject 2 had to be dropped. The selected model achieved RMSE of 2.298 deg/s and Pearson Correlation of 0.961 on the pre-training group data. However, on the validation group data, the selected model achieved RMSE of 4.701 deg/s.

In Table 1, RMSE and Pearson Correlation for all new individuals are presented for each task. When tested on eight new participants, the average RMSE increased and the Pearson Correlation decreased, which means the performance drop of the model by unseen data. However, if the “Quick training” process was applied with a small amount of data, the results improved to similar level as those of the training group. For example, in the Bulb task, the selected model had RMSE of 19.723 deg/s, and a Pearson Correlation of 0.669 on the training group. If new participants were applied to the model, RMSE increased to 43.977 deg/s, and Pearson Correlation dropped to 0.526. After the “Quick training” process was utilized, the average RMSE decreased to 25.813 deg/s and the average Pearson Correlation rose to 0.702. Similar trends were also observed through other tasks. In general, the performance improved after the “Quick training” process, however, there were some exceptional cases, especially on the Cup task for participants 1 and 6. This discrepancy of performance between the participants will be further discussed in the following section.

**Table 1:** RMSE^*^ and Pearson's correlation (PC) values between measured and predicted angular velocity of regression module before and after Quick training.

**Task**	**Train**	**Metric**	**1**	**2**	**3**	**4**	**5**	**6**	**7**	**8**	**Ave**
Bulb	WO	RMSE^*^	104.09	20.96	33.66	34.07	40.47	148.33	33.27	41.32	43.98
	Quick	PC	0.30	0.60	0.66	0.80	0.50	0.01	0.85	0.61	0.53
	With	RMSE^*^	30.31	21.28	27.56	21.15	35.56	22.26	26.78	21.62	25.81
	Quick	PC	0.73	0.62	0.68	0.78	0.71	0.77	0.66	0.67	0.70
Screw	WO	RMSE^*^	50.25	26.97	30.26	48.45	172.91	12.05	62.53	25.70	53.64
	Quick	PC	0.65	0.71	0.48	0.32	0.36	0.82	0.54	0.64	0.57
	With	RMSE^*^	15.49	20.23	20.49	20.71	31.60	8.19	10.25	14.90	17.73
	Quick	PC	0.77	0.85	0.60	0.59	0.73	0.82	0.68	0.68	0.72
Hammer	WO	RMSE^*^	10.05	5.13	3.31	3.81	6.77	9.56	10.24	4.61	6.68
	Quick	PC	0.36	0.41	0.16	0.15	0.48	0.21	0.22	−0.08	0.24
	With	RMSE^*^	8.43	6.38	2.68	3.70	6.37	2.90	7.39	3.98	5.23
	Quick	PC	0.48	0.54	0.38	0.19	0.49	0.42	0.33	0.06	0.36
Cup	WO	RMSE^*^	13.02	–	3.15	9.79	7.29	4.42	14.20	3.59	7.92
	Quick	PC	−0.15	–	0.54	0.45	0.57	−0.13	0.68	0.21	0.31
	With	RMSE^*^	6.23	–	3.06	9.36	7.13	4.33	14.47	3.23	6.83
	Quick	PC	−0.22	–	0.59	0.45	0.57	−0.21	0.66	0.26	0.30

A total of eight new participants were tested. ^*^Unit of RMSE is deg/s.

## 5. Discussion

The presented study shows a new framework to use real ADL task data to train a multi-DoF prosthe tic wrist using sEMG signals. The “Quick training” shows the utilization of a large data pool for creating a generic model but applies to a new user by using only a small amount of data for improving the model performance. Four tasks were tested to create the ML models by recruiting a total of 24 participants and tested on eight participants, which showed comparable performance with other models using a larger data set or training only simple motions.

Comparing between tasks, the Screw and Bulb tasks showed higher Pearson‘s correlation than the Cup and Hammer tasks. This is presumably because high variation was found in the movement in Cup and Hammer tasks for various reasons. First, participants chose different movement strategy to perform the Cup and Hammer tasks. Some participants preferred moving only their wrists when lifting the hammer, while other participants preferred only moving their wrists when dropping the hammer. Participants chose different movement coordination between the wrist, elbow, and shoulder to perform the Cup and Hammer task. Second, the end-effector (tool) movement to fulfill the task had different kinematic redundancy. The Bulb and Screw tasks required to rotate the screw or bulb exactly along the screw thread. However, the cup or hammer task was not performed with restricted end-effector as Bulb and Screw tasks. Lastly, participants had different fluency to perform the hammer task. Even though 5-min practice session was provided for each task, there were participants who never used a hammer before. This could be another factor to create deviation in the movement, resulting different sEMG patterns among participants. Even though higher Pearson's Correlation was observed in the Cup and Hammer tasks, it should be noted that the Cup and Hammer tasks had larger RMSE. This was due to the different range of motion of the pronation-supination and the dart-throwing-motion tasks. Pronation-supination tasks (Bulb and Screw) had a significantly larger range of motion than dart-throwing-motion tasks (Hammer and Cup), which naturally led to larger RMSE despite higher Pearson's correlation.

A few other researchers also studied various regression models for controlling prosthetic wrist. Stival et al. (2018) combined sEMG and IMU features to control prosthetic systems, and tested their model on a publicly available database as shown in Table 2. The Pearson's correlation of our study in Table 1 was changed to correlation coefficient similar to the study in Stival et al. (2018). Our controller performed comparably to theirs on the Bulb and Hammer tasks, and significantly better on the Screw task, exceeding their sEMG and IMU data fusion methods. It should be noted that Stival et al.'s method only showed results for two tasks that performed the best (three-finger flexion and wrist flexion), while our method focused on more complex ADL movements. Our model was trained with data from 16 participants, with each of them performing three trials, whereas Stival et al.'s method was trained on 35 participants, with six trials each.

**Table 2:** Correlation coefficient for the considered movements Stival et al. (2018) method and ADL tasks in our method.

	**Movement 3**		**Movement 13**	
sEMG and IMU (Stival et al., 2018)	0.7659		0.8634	
	**Bulb**	**Screw**	**Hammer**	**Cup**
Our method	0.7651	0.8863	0.7578	0.5463

Pearson's correlation in Table 1 is converted to correlation coefficient.

Bao et al. (2021a) also proposed a CNN-LSTM model for wrist kinematics estimation. The data was collected from six participants with 12 sensors. Bao et al.'s method trained a model on 3/4 of the data and tested it on the remaining 1/4. The trained model was evaluated by using *R*^2^, and the detailed numeric results for the model are listed in Table 3. Although our method showed less *R*^2^ values, it is important to note that our study performed more complicated ADL movements with only eight sensors. Additionally, our “Quick Training” process required much less training data, and the performance of LSTM models would decrease substantially over time due to its natural instincts that the model itself depends on its previous predictions, which means minor turbulence could cause large deviation. Moreover, the way they combined CNN and LSTM required separate tuning, which would affect the efficiency of the proposed method significantly.

**Table 3:** Best *R*^2^ of the hybrid CNN-LSTM model (Bao et al., 2021a) on single-Dof tasks and our method on ADL tasks.

	**Task**	***R* ^2^**
CNN-LSTM (Bao et al., 2021a)	Flexion/Extension	0.89
	Pronation/Supination	0.70
	Radial/Ulnar deviation	0.83
Our method	Bulb	0.530
	Screw	0.685
	Hammer	0.195
	Cup	0.405

Pearson's correlation in Table 1 is converted to *R*^2^.

Another study proposed the regression Supervised Domain Adaptation (SDA) for estimation of the wrist angle of flexion/extension through sEMG data (Bao et al., 2021b). Domain shifting problem was applied to the model to increase the performance on new subjects. Eight participants were recruited in total, trained on 7, and tested on the last one. The model was evaluated by Normalized root mean square error (NRMSE) and the RMSE of our result in Table 1 was changed to NRMSE for selected models. Detailed information is shown in the Table 4. The study showed that the model had NRMSE of 0.181 on designated simple flexion/extension movements. Our method had slightly worse NRMSE on Bulb (0.191) and Screw (0.185) tasks but achieved further improvements on overall more complicated movements with the introduction of the “Quick Train” process (0.133 on Bulb task, 0.120 on Screw task, 0.138 on Hammer task, and 0.191 on Cup task, respectively).

**Table 4:** Average NMSE of regression SDA (Bao et al., 2021b) on the selected movements and our method on ADL tasks.

	**Flexion/Extension**			
Regression SDA (Bao et al., 2021b)	0.181			
	**Bulb**	**Screw**	**Hammer**	**Cup**
Without “Quick training”	0.191	0.185	0.726	0.443
With “Quick training”	0.133	0.120	0.138	0.191

RMSE in Table 1 is converted to NMSE.

Our future studies will focus on addressing the current limitation of the study. First, we performed four different ADL tasks in the present work, thus, more diverse ADL tasks could be explored, and taking extra data into consideration would potentially improve the performance, such as including elbow angles as additional data when predicting wrist angles for tasks that showed different coordination between wrist and elbow joint movements among participants. Secondly, we used MSE as loss function in our model. The model could be presumably improved by modifying the loss function by introducing functions related to Pearson's correlation. Thirdly, the current model was designed for each task. Future models will classify motions into DTM or PS movements and then performing regression could allow our method to be used more generically, similar to previous work (Swami et al., 2021). Some other promising aspects of model generalization including associating not only types of ADL tasks, but also grasp types (Masiero et al., 2023), or arm positions (Gloumakov et al., 2022), could also be utilized to improve the performance. Lastly, complex ADLs that include three dimensional wrist motion will be trained in the model as well in the future. The current study uses ADLs that focus on majorly one dimensional rotation. In the future, the suggested controller will be implemented in the UBArm (Kim, 2022) featuring all three dimensional rotation of the prosthetic wrist with power grasping. With the UBArm, the tasks that were used to train in the presented paper and new tasks will be evaluated in real-time. To test the controller on amputee participants, the protocol will be further optimized and tested. For example, the number of sensors with less importance will be reduced by computing feature importance. Local surrogate models for identifying feature importance will be used such as SHAP (Lundberg and Lee, 2017) and LIME (Ribeiro et al., 2016a,b) to determine the important sensors. For the amputee participants, the sEMG signals can be inconsistent depending on the location of the amputation. We will test 20% or 30% MVC (Maximum Voluntary Contraction) test and check which position of the muscle shows the most consistent sEMG signals for the controller.

## 6. Conclusion

This study employed a data collection approach that included activities of daily living to ensure the datasets reflect realistic wrist motions used in day-to-day scenarios. A CNN model based on the Inception-Time architecture was implemented to train the models using a specific method that allows the designed wrist controller to perform on new subjects. The Quick training process improved the performance of the controller when facing new subjects, while significantly decreasing on-site training time. We believe our method will provide a practical solution for new participants using the model as well as handling situations where sensors may displace during daily living, muscles can become fatigued, or sensors can become contaminated (e.g., due to sweat).

## Ethics statement

The studies involving humans were approved by Institutional Review Board of University at Buffalo. The studies were conducted in accordance with the local legislation and institutional requirements. The participants provided their written informed consent to participate in this study. Written informed consent was obtained from the individual(s) for the publication of any identifiable images or data included in this article.

## Author contributions

JK: conceptualization and supervision. ZM: data collection and analysis. ZM and JK: visualization, writing the manuscript, and editing. All authors have read and agreed to the published version of the manuscript.
